# Some Genetic and Environmental Effects on Equine Asthma in Polish Konik Horses

**DOI:** 10.3390/ani11082285

**Published:** 2021-08-03

**Authors:** Alicja Borowska, Daria Wolska, Artur Niedzwiedz, Hieronim Borowicz, Zbigniew Jaworski, Marta Siemieniuch, Tomasz Szwaczkowski

**Affiliations:** 1 Division of Horse Breeding, Poznan University of Life Sciences, 60-637 Poznan, Poland; alicja.borowska@up.poznan.pl; 2 Department of Genetics and Animal Breeding, Poznan University of Life Sciences, 60-637 Poznan, Poland; daria.wolskaa@gmail.com; 3 Department of Internal Medicine and Clinic of Diseases of Horses, Dogs and Cats, Wroclaw University of Environmental and Life Sciences, 50-366 Wroclaw, Poland; artur.niedzwiedz@gmail.com (A.N.); jerryhorsevet@gmail.com (H.B.); 4 Department of Horse Breeding and Riding, University of Warmia and Mazury, 10-719 Olsztyn, Poland; zbigniew.jaworski@wp.pl; 5 Department of Reproductive Immunology and Pathology, Institute of Animal Reproduction and Food Research, Polish Academy of Sciences, 10-243 Olsztyn, Poland; m.siemieniuch@pan.olsztyn.pl; 6 Research Station of the Institute of Animal Reproduction and Food Research, Polish Academy of Sciences, in Popielno, 12-222 Ruciane-Nida, Poland

**Keywords:** horse, inbreeding depression, local breed, logistic regression

## Abstract

**Simple Summary:**

Equine Asthma (EA) is a blanket term covering inflammatory diseases of the lower airways in horses. It includes mild-to-moderate equine asthma, which affects horses of any age, and severe equine asthma, which is typically seen in horses older than 7 years of. Relationships of the disease’s occurrence with sex or breed have been proven. However, some authors consider genetic background a predisposing factor, due to the fact that in some bloodlines a clinical form of asthma is more frequently observed. This indicates serious breeding and economic consequences. This study aimed to identify the factors affecting predisposition to severe equine asthma in the population of Polish Konik horses and some environmental and inbreeding effects on the disease. Generally, in the observed population, EA is negligibly affected by the factors analysed. Individual inbreeding effects on asthma were not confirmed by various statistical approaches, but significant maternal inbreeding effects were observed. These results are very important from the perspective of the currently implemented genetic resource conservation programme.

**Abstract:**

Current knowledge of the genetic and environmental backgrounds of equine asthma seems to be insufficient, especially for primitive horse breeds. The main objectives of this study were to estimate the effects of sex, birth period, stud, parentage line and inbreeding on asthma morbidity in Polish Konik horses. Records of 274 horses (housed in two studs) were analysed. These animals were allocated to maternal and paternal lines. Individual inbreeding coefficients were extracted from the additive relationship matrix. Horses underwent diagnosis based on observation of the basic symptoms (high frequency of coughing and excessive nasal discharge). Subsequently, some horses (28 individuals) were clinically examined to confirm the earlier observations. Generally, no significant effects of parentage line on heaves morbidity were identified by the use of logistic regression, although the Pearson’s chi-squared test had shown that individuals of some maternal and paternal lines had a predisposition to severe equine asthma. It was concluded that the individual inbreeding level is not associated with the incidence of EA, but a significant effect of the maternal inbreeding coefficient may be observed. It was also found that there is some variability in the incidence of this disease between studs.

## 1. Introduction

Polish Konik horses can be regarded as a living part of European cultural heritage. The breed is a native one, directly derived from the wild horses called Tarpans (*Equus ferus*), which became extinct in the 18th century [1,2,3]. The Polish Konik has been conserved since 1936, one of the first breeds of horses in the world to have such a status [4]. Initially, genealogical documentation included 35 maternal lines and 6 paternal lines, but presently only 16 maternal lines are included in the Polish Konik Conservation Programme [5]. Those Polish Konik horses which serve to maintain the purity of the breed are additionally endangered, as they are exposed to an uncontrolled increase in the level of inbreeding. This can considerably reduce the genetic variation, performance, reproduction and survival of the animals [6,7,8].

Respiratory system diseases in horses are a considerable problem for both breeders and riders. After disorders of the musculoskeletal system, they are the second most important group of ailments that cause problems with physical effort, leading to exclusion from breeding and sports [9,10]. In young animals, the predominant syndromes include diseases of the upper respiratory tract and lung afflictions caused by infectious agents. In horses older than 5 years, the most common reason of morbidity is allergy [11].

Equine Asthma (EA) is a blanket term covering inflammatory diseases of the lower airways in horses. It includes mild-to-moderate equine asthma, which affects horses of any age, and severe equine asthma, which is typically seen in older horses.

The pathophysiology of mild-to-moderate equine asthma has not been fully elucidated and is thought to be influenced by both environmental, as well as some genetic factors [12]. Severe equine asthma, formerly also known as recurrent airway obstruction (RAO), and earlier as chronic obstructive pulmonary disease (COPD), is an ailment associated with abnormal functionality of the respiratory system in horses. Presently, around 10–20% of animals over the age of 5 suffer from asthma; however, the number of such horses is increasing year by year [13]. Genetic predisposition to severe equine asthma was described by Schaeper [14], Koch [15] and Marti et al. [16]. Some authors [17] have hypothesised a polygenic inheritance model for asthma. On the other hand, Gerber et al. [18] reported the presence of single loci determining this disease (two families of Warmblood horses were studied). Swinburne et al. [11] identified 15 quantitative trait loci (QTL) on eleven chromosomes based on a whole-genome scan in Warmblood horses. The most important QTL regions were identified on ECA13 and ECA15. Some of these genes are responsible for the regulation of T-cells (IL-27) or associated with inducing allergies by inhalation (IL-7R) in human asthma development [19]. A number of reports on clinical aspects of severe equine asthma are available [20,21], including the search for non-invasive blood or urine biomarkers [22]. Equine asthma is well known as an animal model of human asthma [23,24].

The impact of environmental factors on equine asthma has been well described [25,26]. However, our current knowledge on the genetic background in local breeds is still limited. The issue of inheriting the predisposition of diseases in small, closed populations under conservation of genetic resources is extremely important. This paper is the first report on the genetic background of asthma in a primitive horse breed.

The objectives of this study are to estimate the effects of sex, inbreeding, maternal and paternal line, birth period and stud on asthma morbidity.

## 2. Material and Methods

The records of 274 Polish Konik horses (194 females and 80 males) were analysed. The horses were housed in two studs located in distant Polish regions: Masuria and Wielkopolska (under a large climatic variation), denoted as A (107 horses) and B (167 horses). The maintenance systems in both studs were similar. Horses in spring/summer/autumn were kept on pastures (24 h a day), in winter in stables (mares and stallions individually, young horses in groups) with access to paddocks for a few hours. However, due to the variability of weather in different regions of Poland, the length of pastures may vary between studs: the start and end of the grazing season are determined, among other factors, by temperature. The recorded animals were born in the years 1970–2015. The horses were divided into two categories—a healthy (control) group and an asthma-affected (study) group. A detailed description of the data is given in Table 1. The control group consisted of 219 horses classified as free of respiratory problems, with no signs such as coughing or nasal discharge. The study group consisted of 55 individuals. The coughing had to appear regularly, indicating the accumulation of mucus in the respiratory tract. Nasal discharge, one of the most characteristic symptoms of heaves, had to be visible in the sick horses. Clinical signs persisted at all times during exposure to specific allergens They were elite mares and stallions belonging to the breeding stock of the above-mentioned studs. Recorded horses are at least five years old [27]. The individuals were classified into five birth periods, corresponding to decades (Table 1). Effects of inbreeding and parentage lines on the incidence of equine asthma were examined. Pedigree data included information on 9345 horses. Recorded individuals belonged to 17 maternal and 7 paternal lines (Table 1). Some of the lines included very small numbers of individuals. Lines with a minimum of 5 observations were included in the analyses.

### 2.1. Clinical Verification

Animals from one stud were clinically verified by veterinarians: 14 healthy (with no signs of coughing) and 14 with asthma symptoms There, horses were randomly selected and allocated to particular groups, based on their history. A detailed description of the applied diagnostic procedures is given by Niedzwiedz and Jaworski [20].

All experimental procedures used in this study were approved by the II Local Ethical Commission for Animal Experiments in Wroclaw (resolutions: 1/2012, 23 January 2012; 65/2014, 23 April 2014).

### 2.2. Statistical Analysis

Prior to the estimation of inbreeding coefficients, the completeness of pedigree information was determined. The complete generation equivalent (*g_e_*) was examined according to the formula given by Sölkner et al. [28]. The average value of the discrete generation equivalent was determined for the whole population. This can be interpreted as the number of generations compared to the entire ancestry, which provides information on the completeness of the pedigree data. The individual inbreeding coefficients were extracted from the additive relationship matrix based on the algorithm described by Sargolzaei et al. [29]. These computations were performed using the CFC software package [30].

The next step involved statistical inference on the seven studied effects: sex, inbreeding, maternal inbreeding, birth period, stud, and dam and sire lines. The Pearson’s chi-squared test was used, since the analysed trait is a binary variable. An assessment of the impact on morbidity was carried out for four classes of inbreeding (0.0%, 0.0–5.9%, 6.0–9.0%, >9.0%) according to quartiles.

A logistic regression model was used in this study to find the relationships of the studied binary variable with sex, birth period, stud, parentage lines, inbreeding and maternal inbreeding as covariates. Models containing various factors were derived, as follows:

(1) $$\mathrm{Model}\;I:\;ln\;\left(p\right)=b_{0}+b_{1}x_{1}+b_{2}x_{2}+b_{3}x_{3}+b_{4}x_{4}$$

(2) $$\mathrm{Model}\;\mathrm{II}:\;ln\;\left(p\right)=b_{0}+b_{1}x_{1}+b_{2}x_{2}+b_{3}x_{3}+b_{5}x_{5}$$

(3) $$\mathrm{Model}\;\mathrm{III}:\;ln\;\left(p\right)=b_{0}+b_{1}x_{1}+b_{2}x_{2}+b_{3}x_{3}+b_{6}x_{6}$$

(4) $$\mathrm{Model}\;\mathrm{IV}:\;ln\;\left(p\right)=b_{0}+b_{1}x_{1}+b_{2}x_{2}+b_{3}x_{3}+b_{7}x_{7}$$

(5) $$\mathrm{Model}\;V:\;ln\;\left(p\right)=b_{0}+b_{1}x_{1}+b_{2}x_{2}+b_{3}x_{3}+b_{4}x_{4}+b_{6}x_{6}$$

(6) $$\mathrm{Model}\;\mathrm{VI}:\;ln\;\left(p\right)=b_{0}+b_{1}x_{1}+b_{2}x_{2}+b_{3}x_{3}+b_{4}x_{4}+b_{7}x_{7}$$

(7) $$\mathrm{Model}\;\mathrm{VII}:\;ln\;\left(p\right)=b_{0}+b_{1}x_{1}+b_{2}x_{2}+b_{3}x_{3}+b_{4}x_{4}+b_{6}x_{6}+b_{7}x_{7}$$

(8) $$\mathrm{Model}\;\mathrm{VIII}:\;ln\;\left(p\right)=b_{0}+b_{1}x_{1}+b_{2}x_{2}+b_{3}x_{3}+b_{7}x_{7}‡$$

where *p* is defined as the probability of the dependent variable equaling the morbidity of asthma, *b*_0_ is the intercept parameter, *x*_1_ is the sex, *x*_2_ is the birth period, *x*_3_ is the stud, *x*_4_ is the maternal line, *x*_5_ is the paternal line, *x*_6_ is described as the individual inbreeding and *x*_7_ is the maternal inbreeding (as liner covariable), $x_{7}$‡ is the classes of maternal inbreeding and *b*_1_–*b*_7_ are regression coefficients indicating the relative effect of a particular variable on the result.

Based on the above equations, the odds ratio (OR) was estimated (the exponentiation of *b*: *exp(b)*). This parameter is defined as the ratio of the probability of asthma morbidity to the probability of being disease-free. The OR compares the risk of an event occurring in one group in relation to the other, indicated as the reference group. OR = 1 means that the odds for the compared groups are equivalent. OR > 1 indicates that the predisposition of the group is higher than in the reference group. For OR < 1, the tendency to suffer from the disease is smaller. The confidence intervals for OR were also derived [31]. This criterion indicates the strength of influence of various parameters on the incidence of asthma, and their statistical significance. The confidence limit was estimated at 95%.

To compare the quality of the set of statistical models, Akaike’s information criterion (AIC) was used [32]. The computations were performed using the SAS Enterprise Guide 7.1 software package [33].

## 3. Results and Discussion

### 3.1. Pedigree Completeness and Inbreeding Level

The parameters of pedigree completeness for the two groups of horses are listed in Table 2. In the studied population, all horses have both known parents. The longest ancestral paths were found for 7 individuals and included 16 generations. This corresponds with other studies for local horse breeds [34,35,36]. The quality of pedigree also depends on the period of occurrence of the particular breed and generation intervals. Due to the 100 years of history of the Polish Konik horses, the parameters for this population are very satisfactory. The completeness of pedigree was evaluated using the complete generation equivalent. According to Maignel et al. [37], this is the best method to evaluate the quality of ancestor information. It is assumed that complete pedigrees should include information on five generations [38]. The average number of generations calculated in the present study for the full set of horses is 6.7.

It should be noted that the high inbreeding level in the Polish Konik horse population corresponds with its genetic structure. This situation results in lower genetic variability, which may affect negative inbreeding effects on fitness traits [39]. Reductions in genetic diversity and, in consequence, the expression of genetic defects are often observed. In the present study, about 95% of horses in the pedigree database are inbred (with nonzero inbreeding coefficient) (see: Table 2). Assessed inbreeding coefficients for all recorded horses ranged from 0 to 28%. The minimum inbreeding coefficient for the recorded animals was 0 (for four individuals with a minimum of two full generations of ancestors). The average *F* for the control group was 7.1%, and for the asthma group it was 7.2%. This is quite a high level compared with those of Warmblood breeds, such as Holstein horses (2.27%) [40] and the Slovak Sport Pony (2.67%) [36]. In some cases, such low homozygosity may result from incomplete pedigree information. The high inbreeding level results from the fact that it is a closed population under conservation of genetic resources, this being a typical situation for populations housed in zoological gardens [41]. Posta et al. [42] estimated the inbreeding level of another primitive breed, Hucul horses, to be currently around 7.5%. Szwaczkowski et al. [5] assessed the average inbreeding coefficient at 9.3% for the total population of Polish Konik horses. This was quite a high value; by comparison, Wolc and Balińska [43] determined that the same parameter for this breed in three Polish studs ranged from 5.1 to 5.9%. The highest level of homozygosity for healthy individuals was 28%, whereas for asthma-affected horses it was 19%. Some fluctuations of homozygosity, with an upward tendency, have been noticeable in recent years, especially for asthma-affected horses.

There are also publications dealing with the issue of maternal inbreeding effects on the health traits of animals [44,45,46]. In the case of populations under conservation genetic resources, it seems particularly necessary to study the effects of individual as well as maternal inbreeding, because they may be differential [45]. The average maternal inbreeding coefficient for all recorded animals was 5.8% (for 37 dams, the value was 0). The average was 5.5% for the control group and 7.2% for the asthma group.

### 3.2. Impact of the Studied Factors on EA

The results of the statistical analysis of sex, birth period, stud, inbreeding and parentage lines, using two approaches, are listed in Table 3, Table 4, Table 5 and Table 6. All of the evaluations performed non-parametric analysis (Table 3), and logistic regression (Table 4) showed no significant effects of inbreeding on asthma morbidity (the *p*-values were 0.707 and 0.922 respectively), but a significant effect (*p* = 0.022) was observed for the maternal inbreeding coefficient in the logistic regression analyses (Model IV). Generally, no significant effects of sex and parentage lines on heaves morbidity were identified with the use of logistic regression, although the Pearson’s chi-squared test had shown significant effects for these factors. Only the analysis for Model III indicated that individuals from some birth periods have a predisposition to equine asthma. Table 7 contains goodness-of-fit statistics for each model. The AIC indicated that Models IV and VIII, including the maternal inbreeding effect, were more adequate.

Several reports on the genetic and environmental backgrounds of EA can be found in the literature. Schaeper [14] was the first to present results on the inheritance of this disorder. According to his study, equine lower airway chronic disease is more frequent in the progeny of affected parents: 14 out of 27 offspring from an asthma-affected stallion were found to suffer from the disorder. More detailed research was performed by Marti et al. [16], who studied predisposition to asthma in two different breeds, Warmblood and Lipizzan horses, with known clinical history of heaves among their ancestors. They demonstrated that offspring with one parent affected by recurrent airway obstruction had a 3.2 times higher risk (*p* < 0.05) of morbidity than animals with healthy ancestors. When both parents were affected by the disease, the risk that the offspring would be asthma-affected was almost 4.6 times higher (*p* < 0.05) than when both parents were healthy.

In the present study, horses from both groups (asthma-affected and healthy) exhibited a similar inbreeding level over the years. To our knowledge, there are no available studies on the effect of inbreeding on asthma morbidity. Generally, the mating of relatives is usually undesirable both for improved and conserved populations. On the other hand, inbreeding effects may be mainly caused by the gene pool of the population. There are many disorders that are revealed at a higher level of homozygosity. Smallbone et al. [47] found the correlation between the inbreeding effect and *Gyrodactylus turnbulli* infection in guppies. The effect of the mating of relatives on lamb survival was estimated by Maxa et al. [48]. However, it is well known that many genetic disorders are not associated with inbreeding. Brault et al. [49] studied the genetic backgrounds of cerebellar abiotrophy in Arabians and found no inbreeding effects on the disease. These parameters were also not found to be associated with atrial fibrillation in Standardbred horses [50].

One-way analysis showed that within the studied maternal and paternal lines there is some predisposition to the disease (Table 3 and Table 4). Estimated odds ratios (Table 5) for the Dzina I maternal line and Liliput paternal line show a higher risk of heaves morbidity than for the reference lines: Zaza and Wicek. It is notable that 80% of descendants from the Dzina I line and 60% from the Liliput line had asthmatic symptoms. Each of these lines accounted for more than 10% of all horses in the affected group. There are few reports describing the influence of breeding lines on asthma morbidity. The predisposition of particular races has not been precisely explained to date. Robinson [51] and Leclere et al. [52] reported no association between heaves and the breed of the horse. On the other hand, Couetil et al. [27] analysed 1444 cases of the disease and reported breed predisposition as one of the risk factors. They suggested that in Thoroughbreds, asthma occurrence was three times higher than in other breeds. In the Polish Konik horse population, the frequency of morbidity of asthma is quite high (25%). Due to their high resistance to the external environment, it would seem that they would be less prone to illness. Polish Konik horses adapt quickly even to harsh external conditions [53]. However, as reported by Sowińska et al. [54], environmental changes considerably affect the welfare and related traits of the horses. It must be recalled that the studied parentage lines were created about 100 years ago [55]. Since then, their genetic diversities have been determined by many factors, including random mating, migration of stallions, and subsequently by selection. The multifactor logistic regression models did not indicate any significant differences in the risk of asthma between parentage lines. However, in the analysis of lines in the models (Table 5), the odds ratios for the Dzina I maternal line and Liliput paternal line are again higher than for the reference lines: Zaza and Wicek. Certainly, in further analyses, a careful examination of this factor is essential; it is also necessary to use a larger number of recorded individuals.

As is known, recurrent airway obstruction is considerably affected by environmental factors. It has been confirmed that hay feeding is a major risk factor for heaves [11,18,56]. Ivester et al. [57] found that even in hay of the best quality, some insects, fungal and inorganic dust are present. Factors such as the use of straw as litter and poor ventilation also increase the number of asthma-affected horses. It should be noted that the studied horses came from two studs, but the horse keeping systems in them were similar; the horses largely spent their time in the pastures. However, the parameters of the microclimate in the stables and the quality of the feed were not monitored, therefore the stud effect was included in the analyses. The results show a significant effect of the stud on the risk of asthma; however, this may be due to the parental lines maintained in the compared herds. Pearson’s chi-squared test indicated a highly significant difference between the number of descendants from respective maternal and paternal lines in the compared studs. In the stud A, there were significantly more individuals from the Dzina I and Liliput lines, for whom the conducted analyses showed a greater susceptibility to asthma.

It would be worthwhile to compare more subpopulations from different habitats, and to analyse the frequency of morbidity considering their genetic backgrounds and environmental conditions.

Generally, it was shown that birth period had no statistically significant effect on the occurrence of the disease (Table 3); only Model III in the logistic regression analysis (Table 4) indicated some significant effects (*p* = 0.02). Over successive years, the level of asthma incidences fluctuated. This suggests that there are no significant changes in the gene pool of the whole population that would lead to a larger proportion of asthma-affected horses. Changes of environment and the keeping of animals in closed buildings affected the manifestation of the disease. However, there are no significant trends, in terms of genetic factors, which could cause the number of asthma-affected horses to increase dramatically from year to year, although the odds ratio analysis indicated some significant effects in terms of higher asthma risk for horses born in the period 2000–2009 (Table 6). Previous studies have considered only the age of the animal and its predisposition to equine asthma [9,58]. An increasing number of morbidity cases has been observed in humans suffering from other disorders associated with allergic diseases [59]. The incidence of hypersensitive reactions may also have increased over the last decades in animals.

Finally, the odds ratio (OR), representing the rate of occurrence of the disease in one group relative to the so-called reference group, revealed certain dependencies of the incidence of heaves (Table 5 and Table 6). Each of the analysed factors was associated with some predisposition to heaves morbidity. However, statistically significant effects were identified for stud A, the 2000–2009 birth period, and the parentage lines Dzina I and Liliput. Moreover, the confidence limit exceeds 1. This indicates the relative risk of developing asthma in these groups. In consequence, a genetic background of asthma may be hypothesised. The results obtained in this study also correspond with conclusions reported for Warmblood horses [11,18,60]. Some predispositions were also identified for maternal inbreeding. The non-inbred group contains a high percentage of healthy individuals (92%), whereas statistical inference for the analysis of the individual inbreeding effects should be performed with caution, due to the absence of non-inbred individuals in the asthma-affected group.

In the case of field-collected data (e.g., epidemiological studies), the accuracy of statistical inference depends on the availability of positively defined individuals. This corresponds to simulation studies (see, e.g., [61], where increasing incidence rates of the disease syndrome are positively correlated with the accuracy of estimated parameters under both linear and threshold models). Moreover, for relatively small samples, homogeneity of the experimental material is desirable. Let us recall that in our study, the symptoms of asthma have been verified by veterinarians. This applied to only 10% of the recorded horses (from one stud, only). However, the observations made by breeders were fully confirmed by clinical studies. As already mentioned, the low frequency of sick animals would make it impossible to perform a statistical inference covering experimental factors, which is important from the breeding point of view. Unfortunately, this number of sick animals is still insufficient to estimate genetic parameters, for instance, heritability coefficients. However, in the case of populations under the genetic resource conservation programs, the role of genetic parameters is smaller than in herds with genetic improvement programs.

On the other hand, the present study suggests a genetic background of asthma, manifested by differences among parentage lines and inbreeding levels.

## 4. Conclusions

The results obtained vary depending on the applied method and statistical model. However, the largest differences were recorded between studs, influenced by both environmental conditions and genetic diversity. Moreover, some other considered effects are significant depending on the statistical methodology. No effects of individual inbreeding on equine asthma were confirmed by various statistical approaches, although maternal inbreeding effects were recorded. It should be noted that the inbreeding effects are determined by the gene pool of a given population.

## Figures and Tables

**Table 1:** Number of individuals per subclass across investigated factors.

Item	Total	Group		
		Control	Asthma Positive	
Birth periods				
1970–1979	28	21		7
1980–1989	73	64		9
1990–1999	70	56		14
2000–2009	68	48		20
2010–2015	35	30		5
Maternal lines				
Białka	10	4		6
Bona	5	3		2
Dzina I	10	2		8
Karolka	60	49		11
Liliputka I	18	13		5
Misia II	10	8		2
Popielica	11	6		5
Tarpanka I	40	38		2
Traszka	12	9		3
Tunguska	9	5		4
Tygryska	7	7		0
Urszulka	24	23		1
Wola	24	21		3
Zaza	29	26		3
Paternal lines				
Chochlik	41	37		4
Glejt I	29	25		4
Goraj	33	25		8
Liliput	17	7		10
Myszak	49	38		11
Wicek	104	86		18

**Table 2:** Pedigree structure and completeness of the population studied.

Items	Total	Control	Asthma
Number of pedigreed individuals	9345	219	55
Number of inbred horses	8794	215	55
Average number of generations	6.7	4.6	4.7

**Table 3:** Results of statistical analyses of studied factors of asthma morbidity.

Studied Effect	*p*-Value ^a^	*p*-Value ^b^
Sex	0.001	0.002
Birth period	0.108	0.131
Stud	<0.0001	<0.0001
Maternal line	<0.0001	0.0005
Paternal line	<0.0001	0.007
Individual inbreeding ^c^	0.707	0.922
Maternal inbreeding ^c^	0.102	0.158

^a^ non-parametric analysis ^b^ logistic regression ^c^ classes of inbreeding.

**Table 4:** Results of statistical analyses of studied factors of asthma morbidity in logistic regression models.

Studied Effect	*p*-Value							
	Model I	Model II	Model III	Model IV	Model V	Model VI	Model VII	Model VIII
Sex	0.5353	0.2748	0.3032	0.2337	0.6358	0.4718	0.6004	0.2826
Birth period	0.0752	0.0626	0.0224	0.0537	0.0634	0.0977	0.1058	0.0530
Stud	<0.0001	<0.0001	<0.0001	<0.0001	<0.0001	<0.0001	<0.0001	<0.0001
Dam line	0.3131	#	#	#	0.2429	0.4576	0.3799	#
Sire line	#	0.9234	#	#	#	#	#	#
Individual inbreeding	#	#	0.494	#	0.1023	#	0.0942	#
Maternal inbreeding	#	#	#	0.0218	#	0.0793	0.0605	0.0223 ^‡^

# not analysed; ^‡^ classes of inbreeding.

**Table 5:** Estimates of odds ratio for predisposition to asthma diseases in logistic regression.

Studied Effect	Odds Ratio
mare	4.083
Birth periods	
1970–1979	1.447
1980–1989	0.561
2000–2009	1.637
2010–2015	0.702
Stud A	38.490
Maternal lines	
Białka	13
Bona	5.778
Dzina I	34.667
Karolka	1.946
Liliputka I	3.333
Misia II	2.167
Popielica	7.222
Tarpanka I	0.456
Traszka	2.889
Tunguska	6.933
Tygryska	<0.001
Urszulka	0.377
Wola	1.3
Paternal line	
Chochlik	0.499
Glejt I	0.802
Goraj	1.476
Liliput	6.584
Myszak	1.335

**Table 6:** Estimates of odds ratio for predisposition to asthma diseases.

Studied Effect	Odds Ratio							
	Model I	Model II	Model III	Model IV	Model V	Model VI	Model VII	Model VIII
mare	1.61	2.186	2.093	2.362	1.437	1.737	1.493	2.151
Birth periods								
1970–1979	0.699	0.538	0.471	0.822	0.607	1.01	0.849	1.57
1980–1989	0.623	0.583	0.514	0.579	0.602	0.664	0.636	0.489
2000–2009	3.792	3.257	3.368	3.186	4.441	3.559	4.127	2.567
2010–2015	0.527	0.577	0.587	0.473	0.76	0.496	0.736	0.402
Stud A	112.834	41.883	47.418	45.719	120.017	112.119	122.421	63.117
Dam lines		#	#	#				#
Białka	1.51				1.219	1.777	1.4	
Bona	0.833				0.669	0.733	0.548	
Dzina I	4.103				6.129	2.763	4.059	
Karolka	3.143				2.855	3.063	2.738	
Liliputka I	1.674				1.801	1.566	1.652	
Misia II	0.183				0.151	0.199	0.163	
Popielica	0.793				0.93	0.642	0.735	
Tarpanka I	0.31				0.239	0.373	0.295	
Traszka	1.672				1.714	2.139	2.332	
Tunguska	0.868				0.767	0.877	0.745	
Tygryska	<0.001				<0.001	<0.001	<0.001	
Urszulka	4.431				4.384	4.03	3.926	
Wola	3.668				3.353	4.213	3.865	
Sire lines	#		#	#	#	#	#	#
Chochlik		0.685						
Glejt I		0.849						
Goraj		1.332						
Liliput		1.676						
Myszak		1.156						
Individual inbreeding	#	#	0.013	#	<0.001	#	<0.001	#
Maternal inbreeding	#	#	#	>999.9	#	>999.9	>999.9	^‡^
0								0.145
0–5.9%								1.316
6–8.9%								2.137

# not analysed, ^‡^ classes of inbreeding.

**Table 7:** Akaike information criteria for applied statistical models.

Model	AIC
I	189.417
II	191.57
III	184.512
IV	178.36
V	188.503
VI	187.702
VII	186.189
VIII	176.394

## Data Availability

Data available on request due to privacy restriction.
